# Dissecting the Illegal Ivory Trade: An Analysis of Ivory Seizures Data

**DOI:** 10.1371/journal.pone.0076539

**Published:** 2013-10-18

**Authors:** Fiona M. Underwood, Robert W. Burn, Tom Milliken

**Affiliations:** 1 Department of Mathematics and Statistics, University of Reading, Reading, United Kingdom; 2 Independent Consultant, Reading, United Kingdom; 3 TRAFFIC International, Harare, Zimbabwe; Universidad Veracruzana, Mexico

## Abstract

Reliable evidence of trends in the illegal ivory trade is important for informing decision making for elephants but it is difficult to obtain due to the covert nature of the trade. The Elephant Trade Information System, a global database of reported seizures of illegal ivory, holds the only extensive information on illicit trade available. However inherent biases in seizure data make it difficult to infer trends; countries differ in their ability to make and report seizures and these differences cannot be directly measured. We developed a new modelling framework to provide quantitative evidence on trends in the illegal ivory trade from seizures data. The framework used Bayesian hierarchical latent variable models to reduce bias in seizures data by identifying proxy variables that describe the variability in seizure and reporting rates between countries and over time. Models produced bias-adjusted smoothed estimates of relative trends in illegal ivory activity for raw and worked ivory in three weight classes. Activity is represented by two indicators describing the number of illegal ivory transactions – Transactions Index – and the total weight of illegal ivory transactions – Weights Index – at global, regional or national levels. Globally, activity was found to be rapidly increasing and at its highest level for 16 years, more than doubling from 2007 to 2011 and tripling from 1998 to 2011. Over 70% of the Transactions Index is from shipments of worked ivory weighing less than 10 kg and the rapid increase since 2007 is mainly due to increased consumption in China. Over 70% of the Weights Index is from shipments of raw ivory weighing at least 100 kg mainly moving from Central and East Africa to Southeast and East Asia. The results tie together recent findings on trends in poaching rates, declining populations and consumption and provide detailed evidence to inform international decision making on elephants.

## Introduction

The illegal ivory trade remains a major threat to elephant populations. There is evidence of increased poaching of elephants for their ivory, from the global monitoring program MIKE (Monitoring the Illegal Killing of Elephants) [1], [2] and from regional, national and site level case studies [3]–[5]. Furthermore, there is evidence of declining populations of elephants in some regions [6], [7] and countries [1], [8], [9]. These studies identify major sources of ivory but do not identify what happens to the ivory once it has been poached. Evidence of global trends in the illegal ivory trade, and the identification of trade route patterns from source to destination are required to provide a better understanding of the trade and to assist in decision making for elephants.

The covert nature of the illegal ivory trade is a serious obstacle to quantitative study of the process. The trade typically has been studied by describing the extent of domestic ivory markets in specific countries [10]–[13] or by focussing on economic models [14], [15], or other approaches [16] which correlate national populations of elephants before and after the 1989 ban on ivory trade, with variables describing various aspects of the illegal trade. However, none of this research provides a global picture or quantitative evidence of global trends in the trade. We show that, using Bayesian statistical modelling, data on seizures of illegal ivory can produce trends and reveal underlying dynamics of the illegal ivory trade.

The Elephant Trade Information System (ETIS) was mandated by CITES (Convention for International Trade in Endangered Species of Wild Fauna and Flora) in 1997 to track the illegal ivory trade globally; it is the sister programme to MIKE. CITES Parties (countries that are members of CITES) are requested to report all illegal ivory seizures to ETIS within 90 days. We use over 11,000 ETIS records from 1996 to 2011 to provide quantitative evidence on trends in the illegal ivory trade, in particular global trends in the total weight and number of illegal ivory transactions. We also identify regions and countries and their roles in the trade.

### Using Seizures Data to Understand the Illegal Ivory Trade

**Figure 1:**
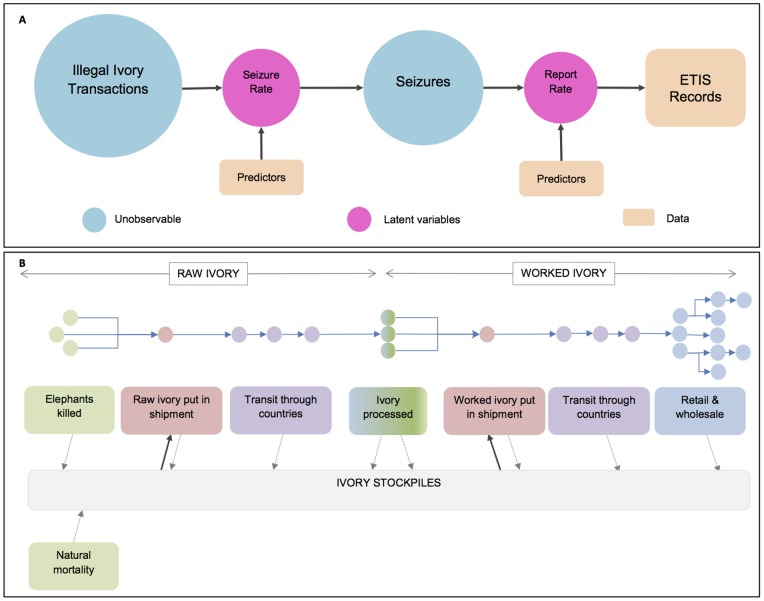
Conceptual models of the illegal ivory trade and seizures data. (A) In each county, in each year an unknown proportion (seizure rate) of illegal ivory transactions (see Figure 1B for examples) is seized. Of these seizures an unknown proportion (reporting rate) are reported to ETIS. We identify potential predictors that discriminate different countries ability to make and report seizures so that we can obtain relative estimates of seizure and reporting rates and the numbers of illegal ivory transactions. Figure S1 shows the predictors of seizure and reporting rate identified by the modelling exercise described in this paper. (B) Flow of ivory from source to point of sale representing different types of illegal ivory transactions. Circles represent individual countries along the trade chain. Raw ivory obtained from illegally killed elephants (or stolen from stockpiles of ivory) is put together into a shipment – this ivory may be sourced from several countries. Shipments might pass through a number of countries before arriving at ivory processing plants. Once the ivory is processed into worked ivory it is put into a shipment (again this ivory may come from several ivory processing plants in different countries and from stockpiles) and could pass through several countries before arriving at wholesale or retail places. Once there, it will likely be sold to individuals, locals or tourists, and could pass through several more countries before reaching its final destination. Seizures can occur all along this trade chain and seized ivory should pass to the ivory stockpiles which also includes ivory from natural elephant mortality in range States. There is evidence that ivory sometimes re-enters the trade chain from unsecured ivory stockpiles [45]. The whole, or parts, of the trade chain for any single piece of ivory could occur in one country, one region or across the globe.

The use of seizures data to provide a reliable picture of illegal ivory trade activity has often been dismissed, [17] for example, because of the obvious biases inherent in the data. However, it is intuitively clear that seizures data hold *some* information about the illegal trade, so rather than ignoring the data altogether, we attempt to identify the sources of bias and account for them in the data analysis. Because ETIS contains reported records of illegal ivory seizures, bias arises from two principal sources (Figure 1A). First, not all illegal ivory transactions within a country are seized; the proportion that is seized, the *seizure rate*, is unknown. Second, not all seizures made by law enforcement bodies are reported to ETIS; the proportion that is reported, the *reporting rate*, is unknown.

If it could be assumed that the seizure rate and reporting rate were the same for all countries and were constant over time, then simple summaries of the ETIS records would be sufficient to describe trends over time, make comparisons between countries and identify those that play a major role in the trade. This would hold even though the actual seizure and reporting rates were unknown. However, there is no *a priori* evidence to justify this assumption, and we proceeded by considering variable rates and the underlying reasons for this variability between countries and over time.

Variation in reporting rate arises from differences both in resources and in the degree of commitment between countries. Each country’s CITES Management Authority (CMA) is the responsible body for reporting to CITES, including the reporting of ivory seizures to ETIS. Some countries have automated systems through which they regularly report to ETIS on all ivory seizure records that were made by their law enforcement agencies. In such cases one might expect that most of the seizures a country makes are reported to ETIS, and the reporting rate to be high. For other countries, reporting to CITES may not be a priority and information on illegal ivory seizures may not come from the CMA, but from NGOs or other sources. Under such circumstances a complete record of all seizures made in that country is unlikely, and the reporting rate is considered low.

Similarly a country’s seizure rate may vary depending on the resources committed to law enforcement. The number of personnel, equipment, training and knowledge of staff would all affect enforcement effort and, in particular, the ability to make ivory seizures. Furthermore, the effectiveness of this enforcement effort may depend on the levels of corruption and governance within the country [18]–[20]. We do not necessarily expect seizure and reporting rate to be related as different agencies are involved in making seizures and reporting these seizures; in general, law enforcement agencies make seizures and CITES authorities report seizures to ETIS. Depending on the degree of inter-agency collaboration, which varies from country to country, two countries with very similar seizure rates may have very different reporting rates.

To capture the variability in reporting and seizure rates, we constructed a statistical model, represented conceptually by Figure 1A. We modelled the seizure and reporting rates in terms of predictor variables representing the causes of variation between countries and over time as described above. Direct observation of the predictors was impossible, so country-specific, time-based candidate proxy variables were sought instead (as listed in Table 1), and those that provided the best fit to the data were identified. Using the best proxy variables the model produced relative bias-adjusted and smoothed estimates of illegal ivory trade activity. The candidate proxy variables, statistical modelling and model selection process are described in the Materials and Methods Section.

**Table 1:** Candidate variables for predictors of seizure and reporting rates.

Description	Source	Proxy for
Corruption Perceptions Index, CPI	Transparency International	LE effectiveness
Control of corruption	World Bank, WGI	LE effectiveness
Government effectiveness	World Bank, WGI	LE effectiveness
Political stability	World Bank, WGI	LE effectiveness
Rule of law	World Bank, WGI	LE effectiveness
Regulatory quality	World Bank, WGI	LE effectiveness
Voice & accountability	World Bank, WGI	LE effectiveness
Gini coefficient	World Bank Poverty Indicators	LE effectiveness
Per capita gross domestic product, GDP	IMF	Economic development
Human development index, HDI	UNDP	Social development
Legislation score	CITES Secretariat	Importance of wildlife crime
LE ratio in previous year	ETIS	LE effort
Data collection score	ETIS	Data collection effort
CITES reporting score	CITES Secretariat	Compliance with CITES reporting requirements

### Characteristics of Illegal Ivory Seizures

Shipments of illegal ivory may be tusks or pieces of tusks, classified as *raw* ivory, or carved or semi-carved pieces of ivory, classified as *worked* ivory. Multiple transactions are typically required to transform a tusk just removed from an elephant into a piece of worked ivory in the home of a consumer (Figure 1B). The trade route of an individual item may be through several countries in shipments of varying sizes, and it could potentially be seized anywhere along this trade chain. For example, an anti-poaching patrol in a protected area may stop poachers carrying a couple of tusks from an elephant they have just killed, border controls may intercept a containerised shipment with a concealed compartment of several tonnes of raw ivory as it moves between two countries to a processing centre, local police may seize worked ivory in a raid on retail outlets illegally selling ivory products, or customs may intercept tourists returning home with pieces of worked ivory in their luggage.

The overall aim of our modelling was to provide estimates of illegal ivory activity over time and the contribution of different countries to this activity. Activity is defined by both the number of transactions and the total weight of these transactions and so from our model we produced two indices. These were a Transactions Index (TI) representing the relative number of illegal trade transactions, and a Weights Index (WI), representing the relative overall weight of ivory in trade. We do not expect trade patterns to be the same for, for example, small pieces of worked ivory in tourists’ luggage and large shipments of raw ivory *en route* to an ivory processing plant. In particular, we might expect the countries involved and the trends over time to differ. We therefore modelled illegal ivory trade activity in six different ivory classes. These classes represent raw and worked ivory separately divided into three weight categories: less than 10 kg (small), 10 kg to less than 100 kg (medium) and 100 kg or more (large). As shown in Figure 1B, raw ivory trade largely reflects the dynamics associated with the movement of ivory from African elephant range states, the principal source of ivory today, to centres of processing that are often elsewhere in the world. Worked ivory trade describes the consumption of ivory as reflected in ivory markets found in both elephant range states and end-user countries, including the tourist trade of worked ivory curios. The transition between raw and worked ivory occurs at ivory processing centres (Figure 1B) and so countries where ivory carving takes place may contribute to both the raw and worked ivory classes depending on whether seizures are made as ivory enters or leaves processing centres. The TI and WI were estimated for each of these six ivory classes, as described in the Materials and Methods Section.

## Results

### Seizure and Reporting Rates

In all, four proxy variables from the list in Table 1 emerged from the model selection process. The seizure rate was found to increase with *LE ratio*, our proxy for law enforcement effectiveness, and with *rule of law,* one of the World Bank’s governance indicators [21]. Several measures of governance and socio-economic development were found to be effective predictors of the seizure rate. However, since these variables were correlated, only the best predictor, *rule of law* was required in the final model. Variability in the reporting rate was described by two variables: the *CITES reporting score*, a proxy for how seriously a country takes reporting to CITES in general; and the internal variable *data collection effort*, a proxy for the effort made by CMAs and ETIS to report and obtain records of illegal ivory seizures. The parameter values for the four variables are given in Table S1 and their place in the conceptual model are shown in Figure S1. Of the four variables that affect seizure and reporting rate, it is the data collection effort that has the greatest effect.

**Figure 2:**
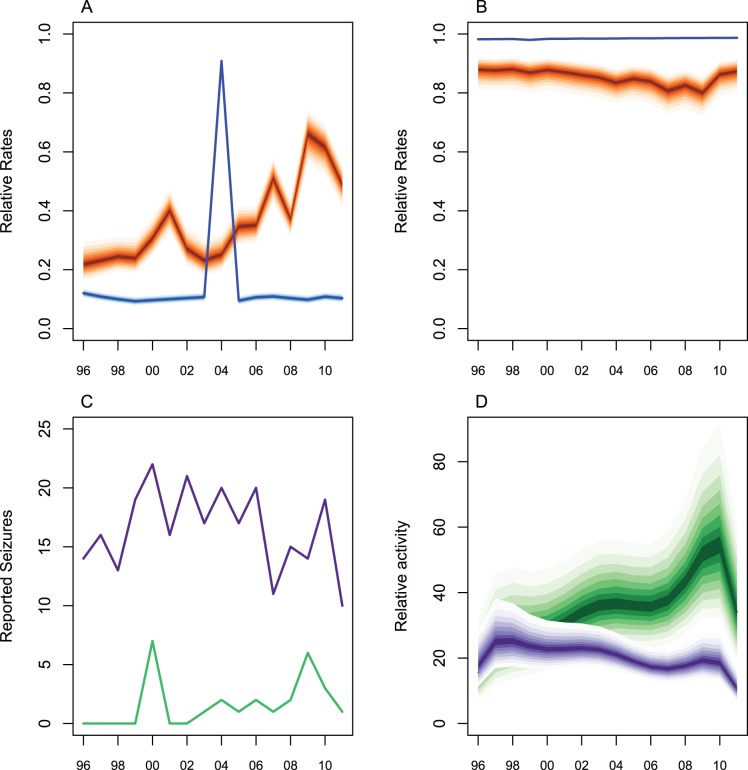
Bias adjusted and smoothed expected number of small raw ivory transactions for USA and Cameroon. (A) Relative seizure (orange) and reporting (blue) rates for Cameroon. Dark shading indicates most likely (median) values and 80% credible intervals are shown. The reporting rate is generally low because reports of illegal ivory seizures are made by NGOs rather than CITES Management Authority (CMA) except in 2004 when the CMA reported to ETIS. Credible intervals for reporting rate are much smaller than for seizure rate. (B) Relative seizure (orange) and reporting (blue) rates for USA. Shading as for A. Reporting rate is high because the USA provides automated and routine reports of illegal ivory seizures to ETIS. (C) Number of reported small raw ivory seizures for USA (purple) and Cameroon (green). (D) Bias-adjusted and smoothed relative numbers of small raw ivory transactions for USA (purple) and Cameroon (green). Dark shading indicates most likely (median) values and 80% credible intervals are shown.

A smooth trend of activity level was derived from a fitted 6^th^ order polynomial of time. The TI was derived from posterior predictive distributions of bias-adjusted and smoothed transactions for each country and year in each ivory class. The bias adjustment has, in some cases, dramatically changed the impression given by the seizures data alone. An illustration of this is to consider Cameroon which had very low seizure and reporting rates (Figure 2A) compared to the USA (Figure 2B). Cameroon reported many fewer small seizures of raw ivory than the USA (Figure 2C). Simply summing these data would suggest that Cameroon had a much less important role in the trade than the USA. After adjusting for the relative seizure and reporting rates in the two countries, Cameroon is estimated to have a similar number of transactions to the USA from 1996 to 1998, and thereafter an increasingly greater number of transactions than the US (Figure 2D). Thus the effect of the bias adjustment is to reveal that the relative roles of these two countries is the opposite of what is suggested by the unadjusted data. The numbers of seizures reported by Cameroon are low because of low reporting and seizure rates not because there is little activity within the country. These two countries demonstrate the extremes and importance of the bias-adjustment effects. The rates for all countries, averaged over the last five years, are shown in Figures S2 and S3. Note the variability in seizure and reporting rates between countries.

### Global Trends

**Figure 3:**
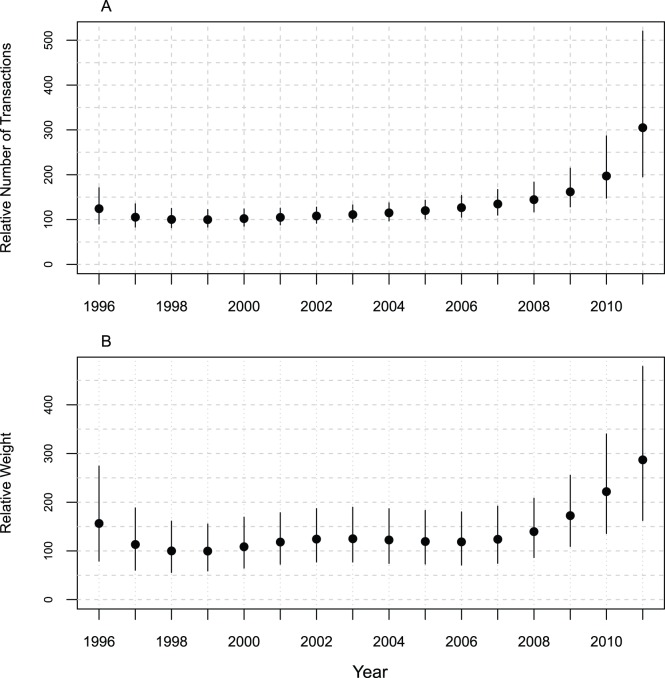
Transactions Index and Weights Index aggregated over ivory classes and countries with 90% credible intervals. (A) Transactions Index (TI) and (B) Weights Index (WI). Dot represents the mean and the line the 90% credible interval for each year. The TI and WI are standardised by setting the 1998 value to 100 to constitute a baseline for comparisons with other years. This year was chosen because it was the first full year after ETIS (and MIKE) were mandated and African Elephant populations in three countries moved from CITES Appendix I to Appendix II enabling a tightly-controlled one-off sale of ivory from these countries to Japan to transpire in 1999.

Illegal ivory trade activity, measured by both the number (TI), and weight (WI) of transactions are increasing year-on-year (Figures 3A and 3B, respectively). For both indices, 2011 levels are approximately three times the 1998 level. The increase was most rapid in the period from 2007 to 2011, when illegal ivory trade transactions more than doubled. Credible intervals are wider for 2010 and 2011 than other years because of the greater uncertainty in these estimates. This is because, firstly, it is likely that the seizures data are incomplete for these two years – for example, some countries that regularly report seizures had not provided complete information for these years. Secondly, there are no data after 2011 to help determine the direction of the trend, unlike years situated in the middle of the time series. Credible intervals for WI are much larger than for TI because of the uncertainty in the weights of seizures (see Data Description for details).

### Trends by Ivory Class and Region

**Figure 4:**
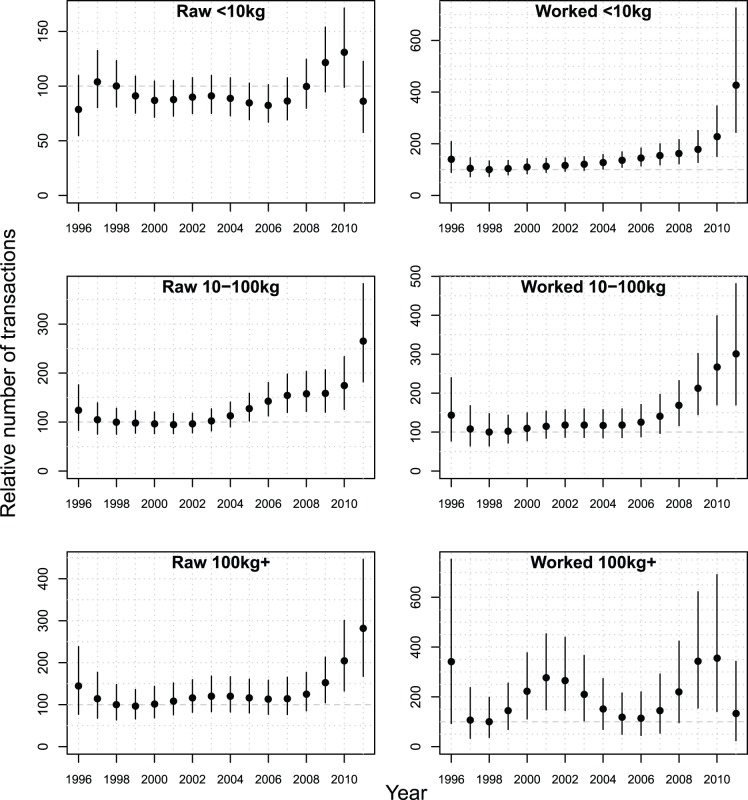
Transactions Index for each ivory class with 90% credible intervals. Dot represents the mean, the line the 90% credible interval for each year. For each ivory class the TI is standardised by setting the 1998 value to 100 to constitute a baseline for comparisons with other years. Note the different y-axes due to the relative increase in each ivory class since 1998.

In all ivory classes there has been an increase in transactions from 1998 to 2010 (Figure 4). With two exceptions, activity continued to increase in 2011. For the exceptions, the small raw ivory and large worked ivory weight classes, both experienced a sharp decline in 2011 following growth from 2007 through 2010. The decrease in small shipments of raw ivory may be because ivory tusks are being aggregated into larger consignments, which would be consistent with the sharp increases seen in the two other raw ivory weight classes. The trends in large shipments of worked ivory have wider credible intervals due to the lower frequency of occurrence in this ivory class, suggesting that processing and retail marketing tends to occur in the same country.

**Figure 5:**
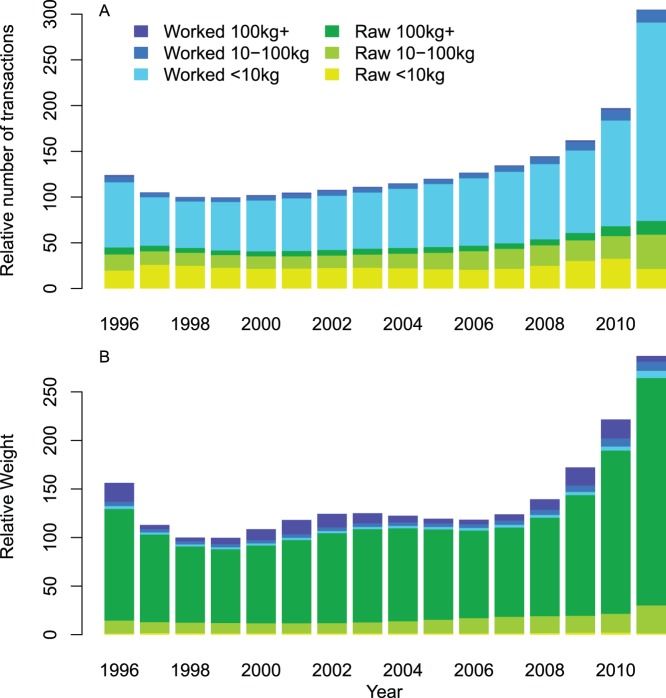
Mean contribution of each ivory class to the Transactions Index and the Weights Index. (A) Transactions Index (TI) and (B) Weights Index (WI) are standardised by setting the 1998 total to 100 to constitute a baseline for comparisons with other years.

**Figure 6:**
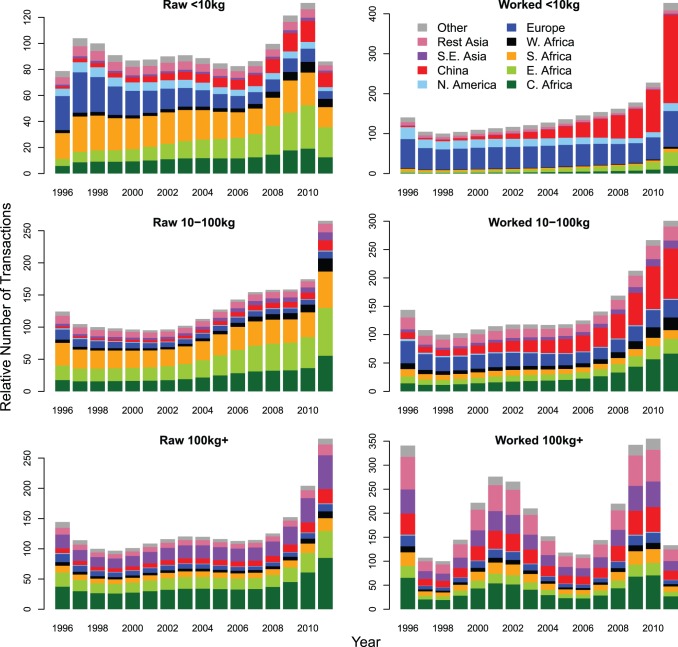
The mean Transactions Index for each region within each ivory class. For each ivory class the Transactions Index (TI) is standardised by setting the 1998 total to 100 to constitute a baseline for comparisons with other years. Mean TI represents the relative number of seizures a country might be expected to make and report to ETIS if seizure and reporting rates were the same over time and across all countries. The countries within each region are listed in Table S2.

In 2011, over 70% of all transactions, and 90% of worked ivory transactions, involved the small worked ivory weight class (Figure 5A). Reflecting demand, in particular end-use consumption, the number and proportion of transactions in this ivory class has increased dramatically over the last three years. The regional breakdown (Figure 6) indicates that Europe and North America were the main consumers in the late 1990s. The substantial increase in this ivory class, however, is due to persistent escalating growth in China’s market, where illicit ivory trade activity in 2011 was more than twice that of Europe and North America combined. The small, but increasing, role of Africa in small worked ivory transactions results from domestic ivory markets in some African countries [22].

Small and medium raw ivory classes are the next biggest contributors to the TI (Figure 5A). For small raw ivory, there has been a steady decline in transactions in Europe and North America when compared to levels in the late 1990s (Figure 6). The early trade largely reflects the use of Europe as a transit route for onward trade to Asia from Africa, but direct trade routes between Africa and Asia have now become the norm. The decline in Europe has generally been matched by an increase in Asian transactions, in particular China. For the medium raw ivory class, consumer regions such as Europe and Asia play a much smaller role suggesting that such trade is either consolidated into larger shipments within Africa prior to export to Asia, or passed to carvers in Africa where the ivory is processed.

Although small worked ivory transactions dominate the TI, it is large consignments of raw ivory that contribute over 70% of the total weight in all years, reaching 82% in 2011 (Figure 5B). Consequently, the sharp increase in the number of transactions in this ivory class is alarming. The relative importance of different regions has not changed greatly over the 16 year period, with Central Africa, Southeast Asia and East Africa accounting for 30%, 20% and 16%, respectively, of the transactions over the last three years (Figure 6). Central Africa and East Africa are both regions with large elephant populations [23], [24], which are under increasing poaching pressure [1], [2], [5], [6], [8] and both regions have major exit ports for illegal consignments of ivory [25]. Large worked ivory consignments contribute about 7% of the total weight of ivory. These consignments are dominated by transactions in Asia (including China and Thailand) and Central Africa, all locations where ivory processing and domestic markets are believed to be some of the largest in the world [22], [25]. More generally, there is evidence of raw ivory being found in Asia, suggesting that ivory processing occurs in countries of transit and destination. There is also evidence of worked ivory being found in Africa suggesting that ivory processing also occurs within Africa and is in fact found all along the trade chain.

### Individual Countries

China has the dominant role of any single country in this analysis: in recent years there has been considerable activity in all weight classes. In the large weight classes, Thailand emerges as the most important Asian country for raw ivory and is second to China for worked ivory. These two nations are currently believed to be the principal end-use markets for ivory, but overall China’s activity is far greater than Thailand’s. Like Thailand, the significant numbers of transactions in the large raw ivory class for Viet Nam and Philippines, and in the large worked ivory class for Taiwan, have increased by about 40% from 2010 to 2011; these locations are thought to act as transit points for onward trade to China and Thailand [25].

In Central Africa, Cameroon plays a major role in all ivory classes. With its seaport Douala, Cameroon has been observed to function as an important entrepôt and exit point for illicit ivory in the past [26]. The Democratic Republic of the Congo, Gabon and Congo all function as major sources of elephant ivory under massive pressure from poaching (3,4,6) and consequently play a major role in large raw ivory shipments. In East Africa, Tanzania, Uganda and Kenya contribute over three-quarters of East Africa’s transactions of large raw ivory. Tanzania and Kenya now function as the key exit points for ivory leaving Africa and Uganda is a regional entrepôt for ivory originating in Central Africa [23].

## Discussion and Conclusions

Our statistical framework has enabled us to quantify trends in illegal ivory trade. We see clear evidence of an alarming, rapid increase in illegal ivory trade activity. This appears to be mainly because of increasing demand in China and Thailand. These end-use markets entail large shipments of raw ivory from Africa, either directly or via other Asian countries. These results tie together recent findings on trends in poaching rates [1]–[5], declining populations, in particular in Central Africa [6], [8], and increasing consumption in East and Southeast Asia [9]–[11] and informed a recent report presented at CITES [27]. Despite the complexity of the modelling our indices provide simple clear summaries of illegal ivory trade activity.

Further insights into the dynamics of the illegal ivory trade can be drawn from our analysis. For example, in the most recent ETIS report to CITES [25], countries implicated in very large shipments of ivory were identified by applying our bias adjustment to seizures over one tonne [28]. A cluster analysis based on these adjustments together with other model outputs identified countries with similar characteristics. This analysis led to a decision by CITES to subject a number of counties or territories associated with consumption (China and Thailand), transit (Philippines, Malaysia, Hong Kong and Viet Nam) and source (Kenya, Tanzania and South Africa) to an oversight process [29].

The analysis presented here describes trends in illegal ivory trade activity and is a prerequisite for an eventual analysis of the drivers of these trends. Of particular concern to CITES is whether two tightly regulated “one-off” sales of ivory in 1999 and 2008 have had an impact on the trade. To understand the impact of these and other CITES decisions, it is necessary to identify hypotheses linking them with trade dynamics. Because CITES decisions are implemented in a constantly changing, complex socio-economic environment, a full causal analysis is required that considers all other potential drivers of ivory trade and their interactions along the trade chain. Without this comprehensive analysis the impact of an individual driver may be confounded with the effects of other drivers.

Although much can be learned from our analysis about the dynamics of the illegal ivory trade, we cannot deduce absolute quantities of ivory in circulation. Even if this were a possibility, there are difficulties in inferring a key quantity of interest to elephant conservationists – the number of elephants killed in a year. Specifically, we currently do not know how long it takes to accumulate a large consignment or how much illegal ivory comes from stockpiles (Figure 1B), some of which is ivory obtained from natural mortality or may have been previously seized and recorded in ETIS.

The methodology developed in this paper has enabled major sources of bias in seizures data to be reduced by identifying key predictors of variation in the seizure and reporting rate between countries and over time. Other predictors may emerge that account for additional variability in seizure and reporting rate, and we cannot claim that our model captures *all* of the known biases. However, our analysis does show that relative seizure and reporting rates can be estimated even though their absolute values are not estimable. Furthermore seizure and reporting rates cannot be assumed constant across countries or over time. Hence, simple summaries of illegal ivory seizures data that do not account for these biases can be misleading in recognising both countries of most concern and trends over time.

We believe that the methodological framework that we have developed for revealing trends and patterns in illicit ivory trade from seizures data has potential for application to seizures of other illicit commodities, such as drugs. Provided that sufficient data on seizures and on predictors of seizure and reporting rates are available, the approach could greatly enhance the value of routine seizure records.

## Materials and Methods

### Data

#### Reported seizures data

All seizure records in ETIS report the country that made the seizure, the year in which the seizure was made and the quantity, in pieces or kilogrammes or both, of raw ivory (tusks or pieces of tusks) and worked ivory (carved or semi-carved pieces of ivory). Only 47% of records reported the weight of the seizure which varied from 0.56 g to 6.3 tonnes. To compare raw and worked weights the Raw Ivory Equivalent (RIE) was calculated by dividing worked ivory weights by 0.7 to account for an average 30% loss of ivory in the carving process [30]. Weights for the remaining 53% of records were estimated using regression models fitted to seizure records that reported both the number and weight of raw and worked ivory [26], [28]. The precision of these estimates, especially for worked ivory, was low. Furthermore the recorded weights for many of the large seizures were only rough estimates – for example “four tonnes” – because the authorities lacked the means to weigh the consignment. In light of these uncertainties concerning weights, we used weight classes rather than absolute weights. The estimated weights were used only to assign seizures to broad weight categories.

In total 88 countries reported making at least one seizure to ETIS in the 16-year reporting period, 1996 to 2011. Many records in ETIS report the countries through which the shipment passed or was destined for, thus implicating other countries in the reported seizures. The purpose of our analysis is to identify trends and countries that play a major role in the trade. A principal objective of the bias adjustment is to correct for countries that report few seizures themselves but are heavily involved in the trade. The criterion for including countries was that they should have made or been implicated in either (1) at least 30 seizures in total over the 16 years, or (2) seizures totalling at least 300 kg over the 16 year period. These thresholds were chosen by inspecting the statistical distributions of total weights and numbers of seizures, for each country, over the entire period. The final dataset included data from eight countries - Angola, Benin, Equatorial Guinea, Cambodia, North Korea, Laos, Senegal and Togo – that reported no seizures themselves. The dataset also did not include records from 28 countries that did not meet the selection criteria; collectively these countries made 40 seizures of raw ivory and 82 of worked ivory from 1996–2011. Table S2 lists the 68 countries included in the analysis.

The final dataset used to estimate the Transaction Index, available in Data File S1, consisted of 3,815 seizures of raw ivory and 8,042 seizures of worked ivory. There were 342 seizures that contained both raw and worked ivory and these were included in the appropriate raw and worked ivory weight classes. A summary of the numbers of seizures in each ivory class is given in Table 2.

**Table 2:** Number of seizures in each ivory class.

Type	Weight class (kg)			Total
	*Small*	*Medium*	*Large*	
	*[0,10)*	*[10,100)*	*[100)*	
*Raw*	2,183	1,346	286	3,815
*Worked*	7,567	429	46	8,042
*Total*	9,750	1,775	332	11,857

To estimate the Weights Index all records that reported the seizure weight were used in a separate modelling exercise, as described below. Of these there were 2,470 of raw ivory and 3,227 of worked ivory. These records are available in Data Files S2 and S3 respectively.

The data used in this analysis were seizure records available in ETIS at beginning of May 2012. For the latter years, in particular 2011, the information was inevitably incomplete for some countries.

#### Variables affecting the seizure rate

It was anticipated that seizure rate would be a function of law enforcement (LE) effort and effectiveness. In principle, LE effort could be measured by data on budgets, personnel, training, equipment, etc. Unsurprisingly, such information is impossible to obtain consistently across all 68 countries required for the analysis. Effectiveness of LE was thought to vary according to the background level of corruption or governance, and also the general level of socio-economic development in the country. The strength of wildlife trade legislation in a country was also thought potentially to be a contributory factor. The approach adopted was to seek indicators for these background variables and then to use past ivory seizures data to provide an indirect measure of LE effort.

Possible candidate proxy variables for LE effort and effectiveness that were tried in the modelling are listed in Table 1. Background indicators of corruption, governance and socio-economic development were obtained from publicly available sources. Indicators chosen to represent governance were the *Corruption Perceptions Index* (CPI) of Transparency International [31] and the World Bank’s *World Governance Indicators* (WGI) [21], of which there are six. Selected development indicators were the *Human Development Index* HDI) from UNDP [32], per capita GDP from the IMF [33] and the *Gini coefficient*, as a measure of inequality, from the World Bank’s Poverty Indicators [34]. Most variables were available for each country in each year and missing values were estimated by interpolation. Time-varying values of the *Gini Coefficient* and *HDI* for each country were not available and so the means of available values for each country were used instead.

We used a simple score from the CITES National Legislation Project as an indicator of the strength of wildlife trade legislation (*leg*). The extent to which the country meets CITES requirements for legislation [35] is measured on a 1/2/3 scale. The higher the score the stronger the wildlife trade legislation.

*LE ratio* for a country in a given year was defined as the proportion of all seizures that a country was involved in that were made by the country themselves. Countries that were implicated in many seizures but did not make and report any seizures themselves had a LE ratio of zero. In the analysis we used the one-year lagged LE ratio, as a proxy for LE effort to represent the situation where last year’s LE ratio represents the environment for the current year.

Data for *CITES legislation score* and *LE ratio* are provided in Data File S1.

#### Variables affecting the reporting rate

Reporting rates could potentially vary according to both the readiness of the country to submit data, and the effort made by the ETIS database manager to obtain it. No direct measures of the former were available, so again proxy variables for it were sought. The key variable used was the *CITES reporting score*, an indicator based on each country’s experience in fulfilling reporting requirements to the CITES Secretariat. The variable was calculated as the number of CITES Annual Reports submitted as a proportion of the number of years the country had been a CITES Party. The idea is that the more seriously a country treats its reporting obligations under CITES (including reporting to ETIS) the closer the score to one.

The effort made by the database manager to collect seizures data varied according to the way that each seizure was reported to ETIS. In principle, according to the recommendation in CITES Resolution Conf. 10.10, a seizure should be reported by the CITES Management Authority (CMA) to ETIS within 90 days of the seizure being made. In practice, however, seizures enter the database in different ways. In the past, certain countries have been subjected to *targeted* data collection in which an ETIS representative visits the country, reviews law enforcement records and collects information on elephant product seizure cases. Although little targeting has occurred in recent years, countries are often *prompted* by mail, e-mail or CITES notifications to submit seizure records. Other records arrive *passively*, i.e. unprompted, some originating from sources other than the CMAs themselves, such as NGOs or other unofficial sources. Some CMAs report to ETIS by sending records that have been collected in the context of national automated systems holding wildlife trade seizure information. In these cases, we might expect that most, if not all, seizures made in that country are reported. In the case of passive, unsolicited reporting it is less clear that all seizures are reported to ETIS. To capture this variability each record in ETIS is scored according to whether it was obtained from targeting or prompting, or from an automated mechanism, or whether it was received passively. We define the *data collection score (DC)* for a country as the proportion of records in a year that came from a targeted/automated/prompted mechanism.

Data for *CITES reporting score* and *Data Collection Score* are provided in Data File S1.

### Methods

Two models were fitted to the data. The principal model was a multivariate Bayesian hierarchical latent variable model [36]–[38] represented conceptually in Figure 1A modelling the number of illegal ivory trade transactions. This model was fitted to the data, the number of seizures from each of 68 countries in each of 16 years (1996 to 2011) in each of six ivory classes. The model allows seizure rate and reporting rate to vary between countries and over time. However, the model does not allow these rates to vary between the six ivory classes. The seizure and reporting rates are latent variables and not completely identifiable [37], [39] so suitable proxy variables, as described above (and listed in Table 1), were identified and tested. Using the best proxies we were able to obtain *relative* estimates of seizure and reporting rates that enabled valid comparisons between countries and years. Thus, separate smoothed and bias-adjusted relative estimates of the number of transactions were obtained for each country and each ivory class over time. Because these smoothed adjusted estimates of illegal ivory transactions are contained within one model, we were able to make comparisons of the relative importance of each ivory class. The outputs of this model were combined with a second model of the weight per seizure to estimate the total weight of transactions in each ivory class over time. The outputs from the two models were used to produce the Transactions Index (TI) representing the relative number of illegal trade transactions, and the Weights Index (WI), representing the relative overall weight of ivory in trade. Details are provided below and the Technical Report [28] provides a very detailed description of the methodology and parameter estimates.

#### Statistical model for seizures data

We let 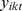 represent the number of reported seizures in country 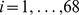, ivory class 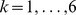 and year 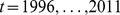. Typically one would start modelling count data like this using a Poisson distribution, but initial exploratory models indicated severe over-dispersion and a negative binomial distribution [40] was therefore used instead.

A key assumption in the model was the factorisation of the mean 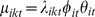, where 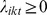 is a measure of the expected number of unobserved class *k* ivory transactions in country *i* and year *t*. 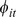 is the seizure rate and 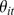 the reporting rate 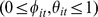 for country *i* in year *t*. Because we wished to make comparisons between ivory classes and because some countries have no reported seizures in some ivory classes, we modelled the mean seizure rate and mean reporting rate across ivory classes.

Variation in 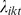 over years was accounted for by fitting a polynomial function of year, *t*, to 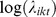. The first two terms, the intercept and linear trend, were allowed to vary across countries, but in order to keep the number of model parameters down to a manageable level, higher order terms were assumed the same for all countries. This simplification was justified by the relatively smaller contribution of the higher order polynomial terms to the overall trend. Polynomial terms up to 9^th^ order were tested.

The country by year specific seizure and reporting rates are latent variables, but were modelled as functions of the candidate proxy variables described above as covariates (predictors). The *logit* link function, 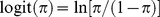, was used to relate them to their linear predictors, so that 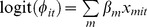 and 
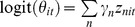
, where 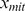 and 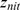 are standardised values of the covariates.

The model was fitted in a Bayesian framework, by MCMC [41] using the OpenBUGS software [42] and the *R* system [43]. Non-informative priors for all parameters were adopted throughout. The full model is given in Text S1. Model selection was based on a combination of DIC [44] and inspection of credible intervals.

The key outputs from the model are the 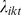 parameter estimates. Their posterior means were interpreted as smoothed and bias-adjusted estimates of the illegal activity in ivory trade – *smoothed* because they are the estimated mean values of a stochastic process, and *bias-adjusted* because the estimated effects of variable seizure and reporting rates have been factored out. The transactions index, *TI*, was calculated as aggregated values (over countries and/or ivory classes as appropriate) of simulations from the posterior distribution of the 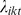 as detailed in Text S1.

#### Estimating the Weight Index, WI

The weight index, WI, was estimated by first interpreting the simulated values of 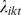, rounded to integer values, as estimates of the number of illegal ivory transactions by country, by year and by ivory class. The weight of each transaction was derived by simulating from the statistical distribution of weight per seizure estimated from seizures with known weights. Raw and worked ivory weights followed different distributions. It was found that the distribution of weight per seizure, for both raw and worked ivory, changed over time. In principle we could have allowed for variation between countries also, but we chose not to do so because of known biases between countries: some countries report weights for those over or under a particular size or do not report weights at all. The model for the distribution of weights was thus a regression model with year as the only predictor variable.

The best fitting model was found to be a robust linear regression of *log(weight)* on year, with residuals following a t-distribution. This model was fitted separately to raw and worked seizures. A Bayesian approach was used to facilitate estimation of the degrees of freedom parameter of the t-distribution. The model is described in Text S1 and parameter estimates of the final model are in Table S3.

For each simulated transaction, a weight was simulated from the estimated distribution of ivory weight per seizure, and then aggregated across countries to get simulated distributions of *WI* for each ivory class *k* and each year *t*. As with *TI*, trend values and credible intervals were derived from these distributions as described in Text S1.

#### Model checking

To check the validity of the models, we obtained posterior predictive distributions [38], [40] of the response variables. For example, for the Transactions Index we obtained the posterior predictive distribution of the number of seizures in each country, year and ivory class. The distribution was compared to the observed 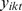 by plotting the mean and 95% credible interval for the poster predictive distribution against the observations over time. Aggregated values for each ivory class and over all ivory classes were also checked.

The model checking identified the inadequacy of describing the number of seizures by a Poisson distribution and a Negative Binomial distribution was more appropriate. The process also identified that an upper limit was required for simulating weights of individual seizures. The final models produced posterior predictive distributions that were compatible with the data. Model comparisons confirmed that a model with separate reporting and seizure rates was more appropriate than a model with a combined seizure and reporting rates.

## Supporting Information

Figure S1 **Conceptual Model of the illegal ivory trade with predictors identified by our modelling exercise.** In each county, in each year an unknown proportion (seizure rate) of illegal ivory transactions (see Figure 1B for examples) is seized. Of these seizures an unknown proportion (reporting rate) are reported to ETIS. Our modelling exercise identified the *lagged law enforcement ratio* and *rule of law* as predictors that discriminate different countries ability to make seizures and the *data collection score* and *CITES reporting* scores as predictors that discriminate different countries ability to report seizures. Using these predictors we have obtained relative estimates of seizure and reporting rates and the numbers of illegal ivory transactions.
(TIFF)

Figure S2 **Relative seizure rates averaged over the last five years for each country.** Dot indicates mean and lines the 90% credible interval for each country. Country codes provided in Table S2.
(EPS)

Figure S3 **Relative reporting rates averaged over the last five years for each country.** Dot indicates mean and lines the 90% credible interval for each country. Country codes provided in Table S2.
(EPS)

Table S1 Posterior means of the coefficients of the standardized predictors for seizure and reporting rates.
(DOC)

Table S2 Regions, ISO country codes and countries.
(DOC)

Table S3 Parameter estimates for weights per seizure model.
(DOC)

Data File S1 **This file contains both the number of seizures for each ivory class for each country from 1996–2011 (summarised in Table S1) and the covariates:** ***CITES legislation score, data collection score, LE ratio, CITES reporting score.*** All other covariates used in the analysis are already in the public domain. These data were used for the main analysis in this paper.
(XLSX)

Data File S2 **Weight (in kg) of each raw ivory seizure and the year in which it was seized.** These data were used to estimate the weights index for the raw ivory classes.
(CSV)

Data File S3 **Weight (in kg) of each worked ivory seizure and the year in which it was seized.** The weights provided in the file are the raw ivory equivalent (RIE) weights obtained by dividing the recorded weight by 0.7 as described in the Materials and Methods section. These data were used to estimate the weights index for the worked ivory classes.
(CSV)

Text S1 **Details of models.**
(PDF)

## References

[1] CITES Secretariat (2012) Monitoring the Illegal Killing of Elephants, CITES CoP16 Doc53.1http://www.cites.org/eng/cop/16/doc/E-CoP16-53-01.pdf.

[2] Burn RW, Underwood FM, Blanc J (2011) Global trends and factors associated with the illegal killing of elephants: a hierarchical Bayesian analysis of carcass encounter data. PLoS One 6(9), e24165. 10.1371/journal.pone.0024165 PMC3166301 21912670

[3] Beyers RL, Hart JA, Sinclair ARE, Grossmann F, Klinkenberg B, et al.(2011) Resource Wars and Conflict Ivory: The Impact of Civil Conflict on Elephants in the Democratic Republic of Congo - The Case of the Okapi Reserve. PLoS One 6(11), e27129. 10.1371/journal.pone.0027129 PMC3212536 22096529

[4] Blake S, Strindberg S, Boudjan P, Makombo M, Bila-Isia I, et al. (2007) Forest elephant crisis in the Congo Basin. PLoS Biol 5(4), e111. 10.1371/journal.pbio.0050111 PMC1845159 17407383

[5] Maingi JK, Mukeka JM, Kyale DM, Muasya RM (2012) Spatiotemporal patterns of elephant poaching in south-eastern Kenya. Wildlife Research 39(3): 234–249 10.1071/WR11017 (2012)..

[6] Maisels F, Strindberg S, Blake S, Wittemyer G, Hart J, et al (2013) Devastating decline of forest elephants in Central Africa. PLoS One 8(3): e59469 10.1371/journal.pone.0059469 23469289 PMC3587600

[7] Bouché P, Douglas-Hamilton I, Wittemyer G, Nianogo AJ, Doucet J-L, et al (2011) Will Elephants Soon Disappear from West African Savannahs? PLoS One 6(6): e20619 10.1371/journal.pone.0020619 21731620 PMC3120750

[8] Bouché P, Mange RNM, Tankalet F, Zowoya F, Lejeune P, et al (2012) Game over! Wildlife collapse in northern Central Africa Republic. Environmental Monitoring and Assessment 184(11): 7001–7011 10.1007/s10661-011-2475-y 22170159

[9] CITES (2012) Elephant Conservation, Illegal Killing and Ivory Trade, CITES SC62 Doc. 46.1http://www.cites.org/eng/com/SC/62/E62-46-01.pdf.

[10] Vigne L, Martin E (2011) Southern China’s illegal ivory trade flourishes. Oryx 45(4): 473–474.

[11] Martin E, Vigne L (2011) The importance of ivory in Philippine culture. Pachyderm 50: 56–67.

[12] Vigne L, Martin E (2010) Japanese demand for ivory declines. Oryx 44(3): 326–327.

[13] Vigne L, Martin E (2008) An increase in demand for ivory items in Ethiopia threatens elephants. Oryx 42(4): 483–483.

[14] Bulte EH, van Kooten GC (1999) Economics of anti-poaching enforcement and the ivory trade ban. American Journal of Agricultural Economics 81(2): 453–466.

[15] Bulte EH, van Kooten GC (1999) Economic efficiency, resource conservation and the ivory trade ban. Ecological Economics 28(2): 171–181.

[16] Lemieux AM, Clarke RV (2009) The international ban on ivory sales and its effects on elephant poaching in Africa. British Journal of Criminology 49: 451–471.

[17] Naylor RT (2005) The underworld of ivory. Crime, Law and Social Change 42: 261–295 (note 64)..

[18] Keane A, Jones JPG, Edwards-Jones G, Milner-Gulland EJ (2008) The sleeping policeman: understanding issues of enforcement and compliance in conservation. Animal Conservation 11: 75–82.

[19] Barrett C, Gibson C, Hoffman B, McCubbins M (2006) The complex links between governance and biodiversity. Conservation Biology 20(5): 1358–1366. 17002753 10.1111/j.1523-1739.2006.00521.x

[20] Smith RJ, Muir RDJ, Walpole MJ, Balmford A, Leader-Williams N (2003) Governance and the loss of biodiversity. Nature 426: 67–70. 14603318 10.1038/nature02025

[21] Kaufmann D, Kraay A, Mastruzzi M (2010) The Worldwide Governance Indicators: Methodology and Analytical Issues, World Bank Policy Research Working Paper No. 5430.

[22] Hunter N, Martin E, Milliken T (2004) Determining the number of elephants required to supply current unregulated ivory markets in Africa and Asia. Pachyderm 36: 116–128.

[23] Blanc JJ, Thouless CR, Hart JA, Dublin HT, Douglas-Hamilton I et al.. (2002) African Elephant Status Report 2002: an update from the African Elephant Database. Occasional Paper of the IUCN Species Survival Commission, No 29. IUCN/SSC African Elephant Specialist Group, IUCN, Gland, Switzerland.

[24] Blanc JJ, Barnes RFW, Craig GC, Dublin HT, Thouless CR et al.. (2007) African Elephant Status Report 2007: an update from the African Elephant Database. Occasional Paper of the IUCN Species Survival Commission, No 33. IUCN/SSC African Elephant Specialist Group, IUCN, Gland, Switzerland.

[25] Milliken T, Burn RW, Underwood FM, Sangalakula L (2012) The Elephant Trade Information System (ETIS) and the Illicit Trade in Ivory: a report to the 16th meeting of the Conference of the Parties. Doc. CoP16 53.2.2, CITES Secretariat, Geneva, Switzerland http://www.cites.org/eng/cop/16/doc/E-CoP16-53-02-02.pdf.

[26] Milliken T, Burn RW, Sangalakula L (2009) The Elephant Trade Information System (ETIS) and the Illicit Trade in Ivory: a report to the 15th meeting of the Conference of the Parties. Doc. CoP15 44.1 Annex, CITES Secretariat, Geneva, Switzerland.

[27] UNEP CITES, IUCN, TRAFFIC (2013) Elephants in the dust – the African elephant crisis. A rapid response assessment. United Nations Environment Programme. GRID-Arendal www.grid.no.

[28] Burn RW, Underwood FM (2013) A new statistical modelling framework to interpret illegal ivory seizures data. A Technical Report describing the new modelling framework for analysing seizures data from the Elephant Trade Information System. Mathematics report series (1/2013) Department of Mathematics and Statistics, University of Reading, UK. www.reading.ac.uk/maths-and-stats/research/maths-report-series.aspx. Last accessed 2013 September 6.

[29] CITES Secretariat (2013) National Ivory Action Plans, SC64 Doc. 2, Bangkok, Thailand, 14 March 2013http://www.cites.org/eng/com/sc/64/E-SC64-02.pdf.

[30] Milliken T (1989) The Japanese trade in ivory: tradition, CITES and the elusive search for sustainable utilisation, in The Ivory Trade and Future of the African Elephant, S.Cobb, Ed. Oxford: Ivory Trade Review Group.

[31] Corruption Perceptions Index (annual updates) Transparency International, Berlin, http://www.transparency.org/research/cpi/overview.Last accessed 2012 May 12.

[32] United Nations Development Program, Human Development Report (2011) UNDP. http://hdr.undp.org/en/reports/global/hdr2011. Last accessed 2012 May 12.

[33] International Monetary Fund (2013) World Economic Outlook Database, http://www.imf.org/external/pubs/ft/weo/2013/01/weodata/index.aspx.Last accessed 2012 May 12.

[34] World Bank Poverty Indicators, http://data.worldbank.org/indicator/SI.POV.GINI.Last accessed 2012 May 12.

[35] Milliken T, Burn RW, Sangalakula L (2002) A report on the status of the Elephant Trade Information System (ETIS) to the 12th meeting of the Conference of the Parties. Doc. CoP12 34.1 CITES Secretariat, Geneva, Switzerland.

[36] Gelman A, Hill J (2007) Data Analysis using Regression and Multilevel/Hierarchical Models Cambridge: Cambridge University Press.

[37] Skrondal A, Rabe-Hesketh S (2004) Generalized Latent Variable Modeling. London: Chapman & Hall/CRC Press.

[38] Lunn D, Jackson C, Best NG, Thomas A, Spiegelhalter DJ (2013) The BUGS Book London: Chapman & Hall/CRC Press.

[39] Catchpole EA, Morgan BJT, Freeman SN (1998) Estimation in parameter-redundant models. Biometrika 84(1): 187–196.

[40] Venables WN, Ripley BD (1994) Modern Applied Statistics with S-PLUS (New York: Springer-Verlag, ed. 2.

[41] Gelman A, Carlin JB, Stern HB, Rubin DB (2004) Bayesian Data Analysis. London: Chapman & Hall/CRC Press, ed. 2.

[42] Lunn D, Spiegelhalter D, Thomas A, Best N (2009) The BUGS project: Evolution, critique, and future directions, Statistics in Medicine. 28: 3049–3067. 10.1002/sim.3680 19630097

[43] R Core Team (2012) R: A language and environment for statistical computing. R Foundation for Statistical Computing, Vienna, Austria. ISBN 3-900051-07-0, http://www.R-project.org.

[44] Spiegelhalter DJ, Best NG, Carlin BP, van der Linde A (2002) Bayesian measures of model complexity and fit (with discussion). J. Royal Statist. Soc. 64(4): 583–639.

[45] Milliken T, Burn RW and Sangalakula L (2007) A report on the status of the Elephant Trade Information System (ETIS) to the 14th meeting of the Conference of the Parties. CoP14 Doc. 53.2 Annex, CITES Secretariat, Geneva, Switzerland. http://www.cites.org/common/cop/15/doc/E15-44-01A.pdf.

