# Supplementary figures and images for: Dissecting the Illegal Ivory Trade: An Analysis of Ivory Seizures Data
Source: PLoS One. 2013 Oct 18;8(10):e76539. doi: 10.1371/journal.pone.0076539 (PMC3799824; doi:10.1371/journal.pone.0076539)

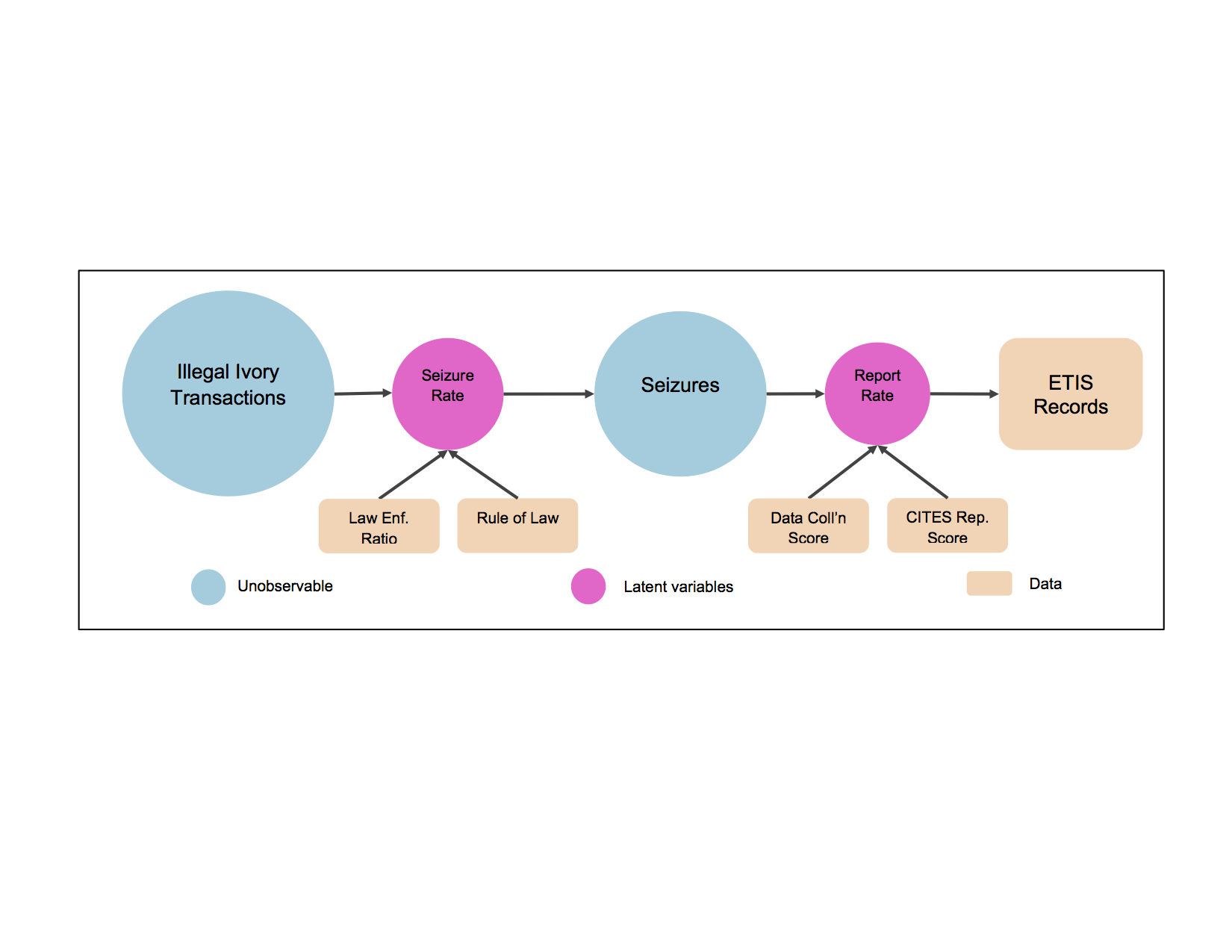

Supplement: Figure S1 — Conceptual Model of the illegal ivory trade with predictors identified by our modelling exercise. In each county, in each year an unknown proportion (seizure rate) of illegal ivory transactions (see Figure 1B for examples) is seized. Of these seizures an unknown proportion (reporting rate) are reported to ETIS. Our modelling exercise identified the lagged law enforcement ratio and rule of law as predictors that discriminate different countries ability to make seizures and the data collection score and CITES reporting scores as predictors that discriminate different countries ability to report seizures. Using these predictors we have obtained relative estimates of seizure and reporting rates and the numbers of illegal ivory transactions. (TIFF) [file pone.0076539.s001.tiff]
