# Supplementary material for: Dissecting the Illegal Ivory Trade: An Analysis of Ivory Seizures Data
Source: PLoS One. 2013 Oct 18;8(10):e76539. doi: 10.1371/journal.pone.0076539 (PMC3799824; doi:10.1371/journal.pone.0076539)
Supplement: Table S1 — Posterior means of the coefficients of the standardized predictors for seizure and reporting rates. (DOC) [file pone.0076539.s004.doc]

# Dissecting the illegal ivory trade: an analysis of ivory seizures data

# FM Underwood, RW Burn, T Milliken

**Table S1: Posterior means of the coefficients of the standardized proxy predictors for seizure and reporting rates.**

| **Variable** | **Predictor (standardized)** | **Posterior mean** | **95% credible interval** |
| --- | --- | --- | --- |
| Seizure rate, | Lagged LE ratio, *LE1* | 0.766 | (0.471, 1.074) |
|  | Rule of law, *rl* | 0.464 | (0.067, 0.864) |
| Reporting rate, $\theta$ | Data collection score, *dc* | 2.521 | (2.246, 2.817) |
|  | CITES reporting score, *rep.sc* | 0.733 | (0.520, 0.949) |

Notes: Since the predictor variables were standardized, it is possible to make comparisons of the numerical values of the coefficients among themselves. We see that the coefficient of the data collection score is much greater than the others, suggesting that the uneven effort made by the ETIS managers to obtain seizures data (and the effort made by the data providers to supply the information) is the major source of bias when attempting to make inferences from seizures records.
