# Supplementary material for: Dissecting the Illegal Ivory Trade: An Analysis of Ivory Seizures Data
Source: PLoS One. 2013 Oct 18;8(10):e76539. doi: 10.1371/journal.pone.0076539 (PMC3799824; doi:10.1371/journal.pone.0076539)
Supplement: Table S2 — Regions, ISO country codes and countries. (DOC) [file pone.0076539.s005.doc]

# Dissecting the illegal ivory trade: an analysis of ivory seizures data

# FM Underwood, RW Burn, T Milliken

Table S2: Regions, ISO country codes and countries

| **Region** | **ISO Code** | **Country** | **ISO Code** | **Country** |
| --- | --- | --- | --- | --- |
| *Central Africa* | cd | Democratic Republic of Congo | cf | Central African Republic |
|  | cg | Congo | gq | Equatorial Guinea |
|  | cm | Cameroon | td | Chad |
|  | ga | Gabon |  |  |
| *East Africa* | et | Ethiopia | sd | Sudan |
|  | ke | Kenya | tz | Tanzania |
|  | rw | Rwanda | ug | Uganda |
| *Southern Africa* | ao | Angola | na | Namibia |
|  | bw | Botswana | za | South Africa |
|  | mw | Malawi | zm | Zambia |
|  | mz | Mozambique | zw | Zimbabwe |
| *West Africa* | bj | Benin | ml | Mali |
|  | ci | Côte d’Ivoire | ng | Nigeria |
|  | gh | Ghana | sn | Senegal |
|  | gn | Guinea | tg | Togo |
| *Europe* | at | Austria | gb | United Kingdom |
|  | be | Belgium | it | Italy |
|  | ch | Switzerland | nl | Netherlands |
|  | de | Germany | pt | Portugal |
|  | dk | Denmark | ru | Russia |
|  | es | Spain | pl | Poland |
|  | fr | France |  |  |
| *North America* | ca | Canada | us | United States of America |
|  | mx | Mexico |  |  |
| *China* | cn | China | mo | Macao |
|  | hk | Hong Kong |  |  |
| *Southeast Asia* | id | Indonesia | ph | Philippines |
|  | kh | Cambodia | sg | Singapore |
|  | la | Laos | th | Thailand |
|  | my | Malaysia | vn | Viet Nam |
| *Rest of Asia* | in | India | kr | Republic of Korea |
|  | jp | Japan | tw | Taiwan |
|  | kp | Democratic People’s Republic of Korea |  |  |
| *Other countries* | ae | United Arab Emirates | eg | Egypt |
|  | au | Australia | nz | New Zealand |
|  | bi | Burundi | qa | Qatar |
|  | dj | Djibouti |  |  |
