# Supplementary material for: Dissecting the Illegal Ivory Trade: An Analysis of Ivory Seizures Data
Source: PLoS One. 2013 Oct 18;8(10):e76539. doi: 10.1371/journal.pone.0076539 (PMC3799824; doi:10.1371/journal.pone.0076539)
Supplement: Table S3 — Parameter estimates for weights per seizure model. (DOC) [file pone.0076539.s006.doc]

# Dissecting the illegal ivory trade: an analysis of ivory seizures data

# FM Underwood, RW Burn, T Milliken

# Table S3: Parameter estimates for weights per seizure model.

|  | **Parameter** | **Posterior mean** | **95% credible interval** |
| --- | --- | --- | --- |
| Raw: | $\delta_{0}$ | 0.308 | (0.247, 0.369) |
|  | $\delta_{1}$ | -0.210 | (-0.270, -0.151) |
|  | $\nu$ | 12.940 | (8.862, 20.020) |
|  | $\sigma^{2}$ | 2.679 | (2.468, 2.900) |
| Worked: | $\delta_{0}$ | 2.366 | (2.299, 2.434) |
|  | $\delta_{1}$ | 0.105 | (0.038, 0.169) |
|  | $\nu$ | 7.917 | (5.950, 10.990) |
|  | $\sigma^{2}$ | 2.193 | (1.990, 2.408) |
