# Supplementary material for: Dissecting the Illegal Ivory Trade: An Analysis of Ivory Seizures Data
Source: PLoS One. 2013 Oct 18;8(10):e76539. doi: 10.1371/journal.pone.0076539 (PMC3799824; doi:10.1371/journal.pone.0076539)
Supplement: Text S1 — Details of models. (PDF) [file pone.0076539.s010.pdf]

Dissecting the illegal ivory trade: an analysis of ivory seizures data

FM Underwood, RW Burn, T Milliken

Supporting Text S1: Details of models

Model for number of illegal ivory transactions

Let y_{ikt} be the number of reported seizures in country $i = 1,...,68$, ivory class $k = 1,...,6$ and year $t = 1,...,16$ and

$$y_{ikt} \sim \text{NegBin}(p_{ikt}, r_k) \text{ where } 0 < p_{ikt} < 1 \text{ and } r_k > 0$$

so that $E[y_{ikt}] = \dfrac{r_k(1-p_{ikt})}{p_{ikt}} = \mu_{ikt} = \lambda_{ikt}\phi_{it}\theta_{it}$ where $\lambda_{ikt} \geq 0$ is the expected number of unobserved class k ivory transactions in country i and year t and $0 \leq \phi_{it} \leq 1$ is the seizure rate and $0 \leq \theta_{it} \leq 1$ the reporting rate. Then we model the number of transactions as:

$$\log(\lambda_{ikt}) = \alpha_{0ik} + \alpha_{1ik}\zeta_1(t) + \sum_{p=2}^{P}\alpha_{pk}\zeta_p(t)$$

where $\zeta_p(t)$ is the pth orthogonal polynomial of year t. The random effects $\underline{\alpha}_{0i}$ and $\underline{\alpha}_{1i}$ are modelled as

$$\underline{\alpha}_{0i} \sim \text{MVN}(\underline{\mu}_0, \Omega_0^{-1}) \text{ and } \underline{\alpha}_{1i} \sim \text{MVN}(\underline{\mu}_1, \Omega_1^{-1})$$

where the Ω's are 6 x 6 covariance matrices. The priors for the μ's and Ω's are non-informative so that

$$\underline{\mu}_0 \sim \text{MVN}(\underline{0}, 10^{-4} I_6) \text{ and } \underline{\mu}_1 \sim \text{MVN}(\underline{0}, 10^{-4} I_6)$$
$$\Omega_0^{-1} \sim \text{Wishart}(R_0, 6) \text{ and } \Omega_1^{-1} \sim \text{Wishart}(R_1, 6)$$

Choosing suitable values for the scale matrices R_0 and R_1 is a little problematic and we adopted the pragmatic approach suggested by Lunn et al (2013). Specifically, we chose $R_0 = \rho\Sigma_0$, where Σ_0 is a prior guess at the covariance matrix and ρ is the degrees of freedom, 6 in this case; R_1 was chosen in a similar way. The values used were

$$R_0 = \text{diag}(50, 50, 5, 300, 75, 25) \text{ and } R_1 = \text{diag}(15, 10, 5, 150, 50, 10).$$

We model the seizure and reporting rates as

$$\text{logit}(\phi_{it}) = \sum_m \beta_m x_{mit} \text{ for } m = 1, \ldots, M$$

$$\text{logit}(\theta_{it}) = \sum_n \gamma_n z_{nit} \text{ for } n = 1, \ldots, N$$

with non-informative priors for the β 's and γ 's so that

$$\beta_m \sim N(0,10^{-4}) \text{ and } \gamma_n \sim N(0,10^{-4})$$

The prior for the degrees of freedom r_k are modelled on a continuous scale, so that DIC can be calculated (Lunn et al 2013), and then rounded.

$$\log(r_k^*) \sim \text{Unif}(0,10) \text{ where } r_k = \text{round}(r_k^*)$$

After convergence, we obtained $\lambda_{ikt}^{(s)}$ for $s = 1,\ldots,5000$ representing 5,000 draws from the posterior distribution of λ_{ikt} by calculating: $\log(\lambda_{ikt}^{(s)}) = \alpha_{0ik}^{(s)} + \alpha_{1ik}^{(s)}\zeta_1(t) + \sum_{p=2}^{P}\alpha_p^{(s)}\zeta_p(t)$

Model for weight of illegal ivory seizures

We let w_{ik^*t} be the weight of ivory of the ith seizure in year $t = 1996,...,2011$ and k^* denotes whether the seizure was of raw or worked ivory. Our model for weight was then

$$\ln\left(w_{ik^*t}\right) = \delta_{k^*0} + \delta_{k^*1}t + \sigma_{k^*}\varepsilon_{ik^*t}$$

The residual $\varepsilon_{ik^*t} \sim \mathrm{t}_{\nu_{k^*}}$ where t_ν denotes the Student t-distribution on ν degrees of freedom.

The prior for the degrees of freedom was uniform so that $\nu_{k^*} \sim \mathsf{Unif}(1,30)$, which allows fractional values and non-informative priors for $\delta_{k^*0}, \delta_{k^*1}$ and σ^2 were used:

$$\delta_{k^*0}, \delta_{k^*1} \sim \mathsf{N}\left(0,10^4\right), \; \sigma^{-2} \sim \Gamma\left(0.001, 0.001\right).$$

After convergence, 5,000 iterations were drawn from the posterior distributions of the parameters and the predicted weights $w_{ik^*t}^{(s)}$ (s = 1, …, 5000) computed from the parameter values: $\log(w_{ik^*t}^{(s)}) = \delta_{0k^*}^{(s)} + \delta_{1k^*}^{(s)}t + \sigma^{(s)}\varepsilon_{ik^*t}^{(s)}, \quad \varepsilon_{ik^*t}^{(s)} \sim \mathrm{t}_{\nu_{k^*}^{(s)}}$.

References

Lunn D, Jackson C, Best NG, Thomas A, Spiegelhalter DJ (2013) *The BUGS Book* Chapman & Hall/CRC Press, London.
